# A Possible Non-genomic Epileptogenic Properties of Estradiol Attenuated by MK801 and DNQX in Amygdala Kindled Rats 

**Published:** 2014

**Authors:** Mehdi Saberi, Fatemeh Saberi, Roshanak Vesali Mahmoud

**Affiliations:** a *Department of Pharmacology and Toxicology, Applied Neuroscience Research Center, Faculty of Medicine, Baqiyatallah University of Medical Sciences, Tehran, Iran. *; b *Department of Pharmacology, Faculty of Medicine, Iran University of Medical Sciences, Tehran, Iran. *; c *Department of Psychology, Faculty of Psychology and Education, Tehran University, Tehran, Iran. *

**Keywords:** Male rats, Amygdalakindling, Seizure, Estradiol benzoate, MK801, DNQX

## Abstract

Although the epileptogenic properties of estrogens have been widely demonstrated in several models and species, the mechanism(s) by which estrogens can acutely change seizure parameters including after discharge and seizure durationremains to be determined. In the present study, we examined the role of NMDA (N-methyl-D-aspartate), non-NMDA andestrogen receptors in estradiol benzoate(EB) effects on kindled seizure parameters.

Different groups of fully kindled male rats received either EB (30 μg /Kg); EB plus MK801 (2 mg/Kg, as NMDA antagonist); DNQX (7.5 mg/Kg);tamoxifen (TAM, 0.1 mg/Kg, as non- NMDA antagonist) or intra-amygdala injection of anisomycine (30 mmol/mL, a protein synthesis inhibitor). Kindled seizure parameters including after discharge duration (ADD) and stage 5 duration(S_5_D) were determined at 0.25 and 3 h post sesame oil (EB solvent) or EB treatment.

While pretreatment with either MK801 or DNQX could block the ADD prolongation induced by EB at 0.25 h, they had no effect on S_5_D prolongation at 3 h. Moreover, application of anisomycine or TAM had no effect on estradiol induced ADD and S_5_D prolongation. These results indicate that both NMDA and non-NMDA receptors could be involved in EB induced ADD prolongation. The observed short termnon-estrogenic receptor or protein synthesis dependent effects of EB may provide a non-genomic mechanism for the stimulatory effects of the steroid on seizure activity.

## Introduction

The stimulatory and epileptogenic properties of estrogens and their role in the catamenial epilepsy are well established elsewhere (1, 12, 15). Also,rapid stimulatory effects of estradiol (E_2_) on kindled seizure parameters have been demonstrated previously in male rats ((14, 19, 20, 21). Although, the mechanisms of these diverse effects are not clear, the amplifying effect of E_2_ on excitatory amino acid activity may play an important role in these complex behaviours. In the classical genomic mechanism taking and lasting hours to days, steroids activate intracellular receptors that regulate transcription and protein synthesis (8, 9, 12). In the more novel non-genomic mechanism, steroids induc very rapid, short-term effects that are more likely due to direct interactions with specific membrane receptors (2, 6, 7, 13, 15, 18, 33). Many of the long-term genomic and short-term membran effects of E_2_(estradiol)can influence synaptic excitatory (3, 25, 30, 31) and inhibitory neurotransmissions (4, 13, 23). Specifically, estrogens augment cerebral purkinje cell responsiveness to iontophoretically applied glutamate (24), alter the sensitivity of neurons to glutamate and NMDA (7, 24, 25, 29) and activate group I and II metabotropic glutamate receptor signalling pathways (2). The activation of glutamate receptors mediate processes involved in the synaptic plasticity associated with learning, memory and epileptogenesis (1). In the hippocampal slice preparation, bath application of E_2_increasesthe extra-cellular CA_1_ neuron field potential in response to the stimulation of the Schaffer collaterals, which makes glutaminergic synapses onto CA_1 _neurons (2, 27). In addition, super-fusion of E_2_inducesa rapid and reversible increment in the amplitude of Scaffercollateral-activated excitatory post synaptic potential (EPSP) in the presence of NMDA antagonist which was blocked by non-NMDA antagonist (31). Also, E_2_potentiatesdepolarization response to glutamate, alpha-amino-3-hydroxy-5-methyl- isoxazole-4- propionic acid (AMPA), kainate and quisqualate (32). Foy and co-workers have demonstrated that oestrogen acts rapidlyvia presumed membrane mechanisms to enhance both NMDA and AMPAreceptor/channel processes in response to glutamate released fromSchaffer collateral terminals (2).These *in-vitro*observations are correlated with the role of both NMDA and non-NMDA receptors in epileptogenic and stimulatory properties of E_2_. 

However, the exactmechanism through which E2 exerts its stimulatory effects in *in-vivo *models of epilepsy such as kindling have not been established yet. The electrical kindling model is regarded as an excellent experimental animal model which is very similar to human complex partial seizures (17). Based on above evidences, in the present study we investigated the role of protein synthesis, NMDA, non-NMDA and estrogenic receptors in observed effects of E2 on kindled seizure parameters in male rats. 

## Experimental

*Animals*

Adult male Sprague-Dawley rats, 250-300 g, were housed in individual cages with unrestricted access to water and purina Rat Chow. The rats were maintained in temperature (about 22 °C) and humidity controlled local animal facilities equipped with 12 hours on (7:00 a.m. to 7:00 p.m.) and 12 hours off light cycle. The study was performed in accordance with the ethical standards and principles of laboratory animal care (NIH publication) and laws of animal protection.

The animals were anaesthetised with ketamine (50mg/Kg) and lidocaine (10 mg/Kg, *i.p*.) and stereotaxically implanted with bipolar stimulating and monopolar recording electrodes (twisted into a tripolar configuration) terminating in the basolateral amygdala of the right hemisphere (co-ordinates: 2.5 mm posterior, 4.8 mm lateral to bregma, and 7.5 mm vertical to dura). The incisor bar was fixed 6 mm above the intra-aural line. Electrodes (stainless steel, Teflon coated, 0.11mm diameter, A-M System, Inc., Carlsborg, USA) were insulated except at the tips. Two other monopolar electrodes were connected to skull screws and placed above the left cortical surface, as earth and differential electrodes, respectively. The electrical kindling procedure was started 10 days after implantation of electrodes. The animals were stimulated daily by 2 second of 60 Hz, biphasic square wave pulses of 0.5 ms per half wave. During the first stimulation session, minimum stimulation threshold (AD threshold) was determined with an ascending series of 25 μA incremental stimulation and 5 minute intervals until at least 5 sec AD recording was achieved as previously described (22). Convulsive responses during kindling were identified using the stages 0-5 paradigms of Racine (17) as follows: Stage 0, no response or motor arrest; Stage 1, facial or jaw movements; Stage 2, addition of head nodding; Stage 3, unilateral forelimb clonus; Stage 4, rearing with bilateral forelimb clonus; and Stage 5, rearing, forelimb clonus and loss of equilibrium. All animals were kindled to 5 consecutive stages 5 seizure before receiving any drugs.

*Drug treatments*

The aim of the study was assessment of the role of NMDA, non-NMDA and estrogenic receptors in EB (estradiol benzoate) effects on ADD (after discharge duration) and S_5_D (stage 5 duration). So to evaluate the effects of MK 801, DNQX) 6, 7- dinitro - quinoxaline - 2, 3-dione( and TAM individuallyon kindled seizure parameters, fully kindled male rats (6-8 animals per each group) were treated with either P.B.S (Phosphate-buffered saline, as drug solvent), MK801 (0.2 mg/Kg, *i.p*.), DNQX (7.5 mg/Kg, *i.p.*, pH=7.4) or TAM (1 mg/Kg) followed by sesame oil (EB solvent, 0.5 mL/Kg) injection after 5 min interval. Kindled seizure parameters were determined at 0.25 h and 3 h post sesame oil injection. Accordingly, the stimulation and recording times were included 0.25 and 3 h post final injection.

After one day recovery, EB (30 μg/Kg, *i.p*.) was applied instead of sesame oil in the above protocol of treatment and kindled seizure parameters were recorded as mentioned above. The doses of drugs were selected as previously described for EB (19, 20), TAM (5), MK801 and DNQX (6).

*Intra-amygdala injection*

Two groups of animals with implanted intra-amygdala electrodes and cannula, received intra-amygdala anisomycine (1 μL of 30 mmol/mL in normal saline solution) through the cannula (29) followed by either sesame oil (control) or EB (test)injection (*i.p*.) after a 5 min interval. Kindled seizure parameters were recorded at 0.25 h and 3 h post the latter injection. All animals were euthanized by diethyl ether anaesthesia at the end of experimental procedure and their brains were removed, sectioned and examined under microscope for electrode tip placement verification.

*Statistical analysis*

The results are expressed as mean ± S.E.M. and statistical significance was evaluated by one way ANOVA*.*Data expressed as percent of control, were compared within and between groups by Wilcoxon andMann–Whitney U-test, respectively. P < 0.05 was taken as significant.

The expressed data as percent of stimulation (AD) threshold were compared within and between groups by non-parametric Wilcoxon and Mann–Whitney U-test, respectively.

## Results

Treatment of animals with either MK801 (0.2 mg/Kg, *i.p*.), DNQX (7.5 mg/Kg, *i.p*.) or TAM (1 mg/Kg) alone had no behavioral changes.

*MK801or DNQX treatment alone*

**Figure 1:**
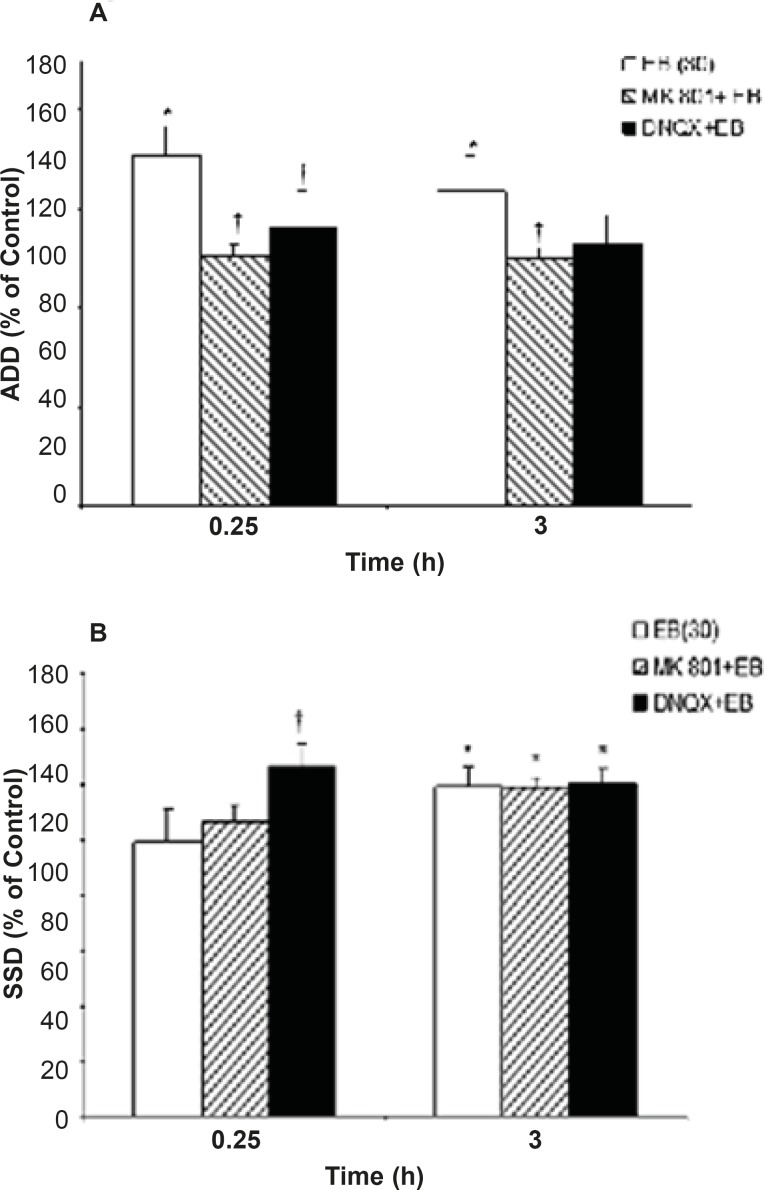
Effects of MK801 and DNQX pretreatment on, (a) afterdischarge duration (ADD) and (b) stage 5 duration (S_5_D) in fully amygdala kindled male rats. MK801 or DNQX were injected (*i.p*.) 5 min prior to estradiol benzoate (EB). Data are expressed as percent of control ± SEM, (control values as seconds is 100% for each group). * or † indicates significant from its control and estradiol benzoate (EB) alone respectively, *P*<0.05, when compared by Mann-Whitney U-test (n=6-8 per group).

EB treatment (30 μg/Kg, *i.p*.) alone was associated with significant increase in ADD (P=0.027) and S_5_D (P=0.043) at 0.25 and 3 h post injection, respectively, when compared to the control group. Administration of either MK801 (0.2 mg/Kg, *i.p*.) or DNQX (7.5 mg/Kg, *i.p*.) alone had no significant effect on kindled seizure parameters in comparison to the respective control values (Figures 1a and 1b).

*Drug pretreatments*

**Figure 2:**
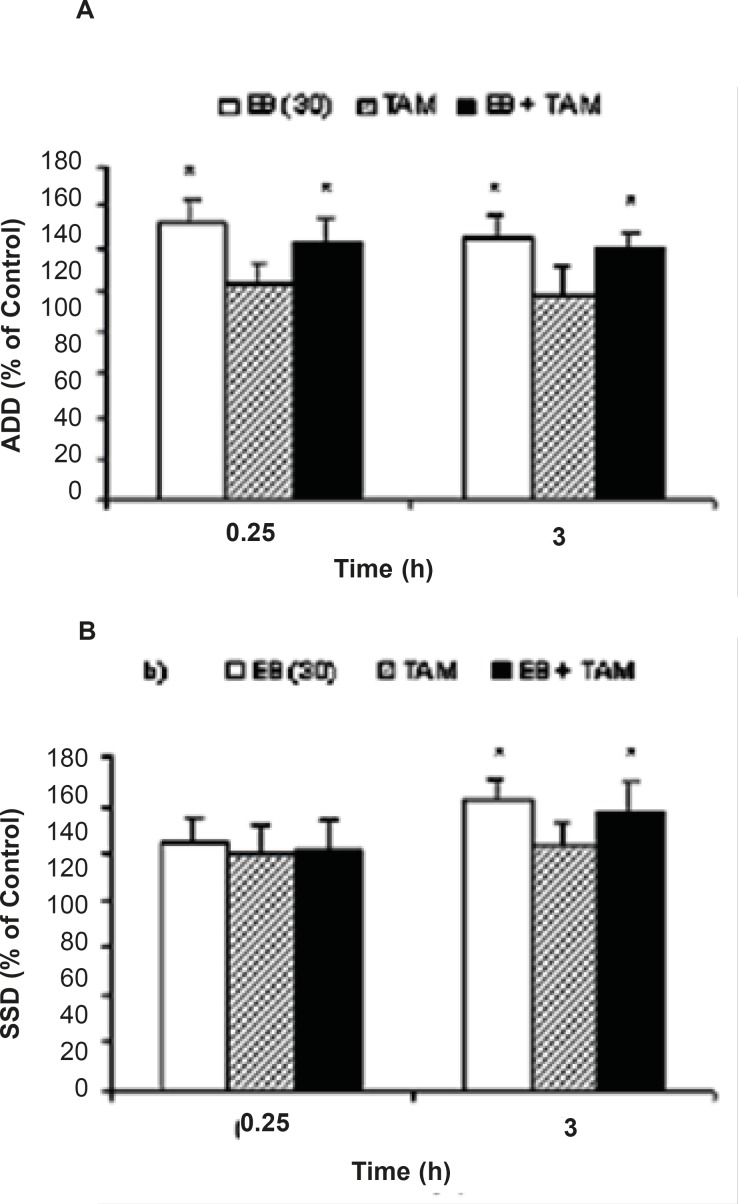
Effects of tamoxifen (TAM) on (a) afterdischarge duration (ADD) and (b) stage 5 duration (S_5_D) in fully amygdala kindled male rats. TAM was injected (*i.p*.) 1.25 h prior to estradiol benzoate (EB). Data are expressed as percent of control ± SEM, (control values as seconds is 100% for each group). * indicates significant from its control and estradiol benzoate (EB) alone respectively, *P*<0.05,when compared by Mann-Whitney U-test (n=6-8 per group).

While EB (30 μg/Kg, *i.p*.) application resulted in a significant increase in ADD, pretreatment of the animals with either MK801 or DNQX could inhibit ADD prolongation (P = 0.042 and P = 0.046, respectively) induced by EB injection at 0.25 h when compared to the EB alone treated group (Figure 1a). However, pretreatment of the animals with DNQX or MK801 could not prevent EB induced S_5_D increment when compared to the EB alone treatment group. On the other hand, the S_5_D increased significantly at 0.25 h (P=0.021) after DNQX pretreatment in comparison to the EB group (Figure 1b).Moreover, pretreatment of animals with TAM had no effect on EB induced ADD and S5D prolongation (Figure 2a and 2b).

*Intra-amygdala anisomycine treatment*

**Figure 3:**
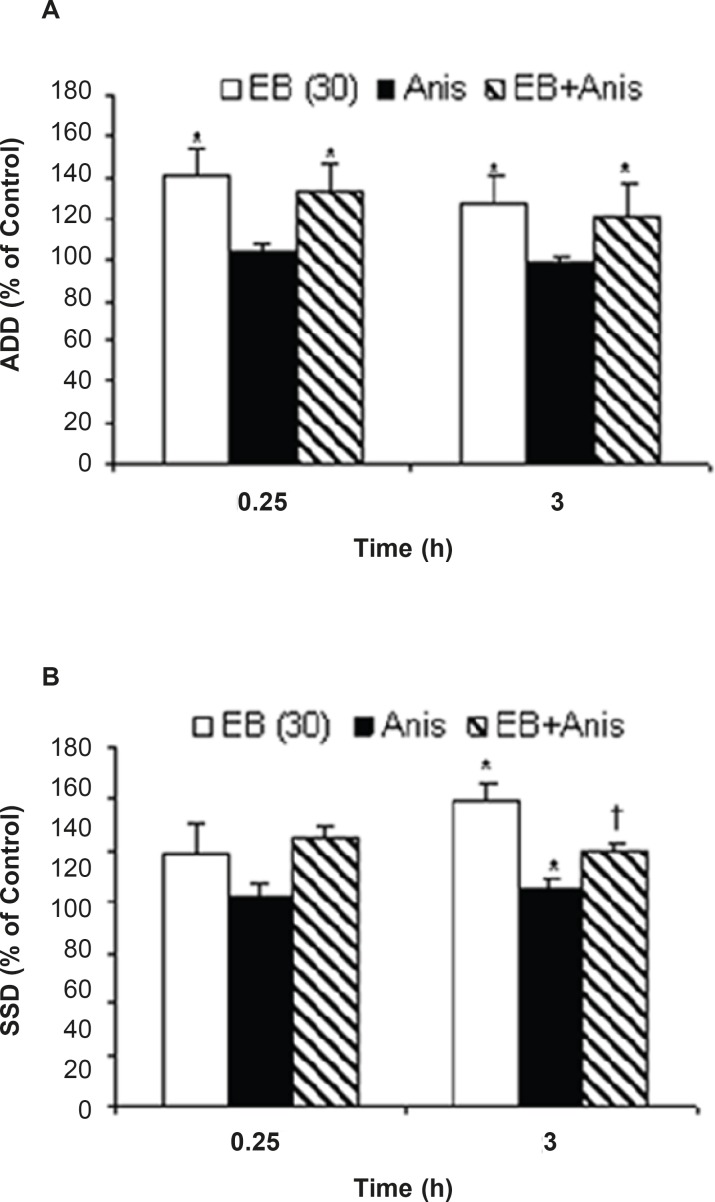
Effects of anisomycine (Anis) pretreatment on; (a) afterdischarge duration (ADD) and (b) stage 5 duration(S_5_D) in amygdala kindled seizure in male rats treated with estradiol benzoate (EB). Data are expressed as percent of control±SEM (control values as seconds is 100% for each group). Each group of animals were pretreated by anisomycine (inter-amygdala injection) 5 min prior to EB administration (*i.p*), and stimulated 0.25 and 3 h after EB treatment. * or †; indicate significant in comparison to respective control and anisomycine alone treated group respectively, *P*<0.05, †; when compared by Mann-Whitney U-test (n=6-8 per group).

Intra-anygdala injection of anisomycine (1 μL of 30 mmol/mL) alone decreased S_5_D at 0.25 (P=0.029) and 3 h (P=0.046) in comparison to the relative control value. Administration of intra-amygdala anisomycine prior to EB treatment was associated with ADD (at 0.25 h, P = 0.038) and S_5_D increment (at 0.25 and 3 h, P = 0.044, P=0.049, respectively) when compared to anisomycine treatment group (Figure3). 

## Discussion

Epileptic disorders, especially in refractory forms, can interfere with the patient’s performance and active presence in the society (28). In many cases, even multi-drug therapy is not effective and in these states patients have to undergo neurosurgical procedures (16). Regarding to the role of estrogens in induction of epileptic activity, determination of the mechanisms involved in this pathway can influence applied medicinal treatments. It had been shown that systemic administration or local application of E_2_ on cerebellum purkinje cell slices could increase significantly the stimulatory response to the glutamate, quisqualate and NMDA rapidly within 5-10 min (6, 11, 33).

In this study, MK801 (a non-competitive NMDA antagonist) pre-treatment inhibited ADD increment induced 0.25 h post EB treatment. However, NMDA antagonist had been more effective against kindling acquisition, but after full kindling the inhibitory effect on stimulation and especially seizure duration was reduced (10). The NMDA receptors are probably more involved in AD propagation and development (10) which is consistent with its involvement in EB effects on ADD increment, as observed in the present study. On the other hand, several reports have stated that E_2_ facilitates non-NMDA receptor activity (14, 26). Wong and Moss (1992) have shown that E_2_ could increase excitatory post-synaptic potential in hippocampal CA_1_ neurons in short- term, which was inhibited by non-NMDA receptor antagonists. Although, E_2_ decreases non-NMDA receptors in several cerebral regions including dentate gyrus (18, 32), however,*in*-*vitro *application of E_2_ on hippocampal CA_1 _neurons increases EPSP within 2 min through non-NMDA receptors. Similarly, the responsiveness to AMPA, kainate and quisqualate had been reported to be increased by E_2 _(32). In the present study, inhibition of ADD prolongation by both receptors antagonists may be an evidence of EB effects on both NMDA and non-NMDA transmissions or their receptor activity. These receptors may play an important role in ADD prolongation probably by acting at different regions of brain. For example, in cerebellum, the NMDA (25) and in hippocampus the non-NMDA (32) receptors participate in EPSP potentiation. The inability of glutamate receptor antagonist to block S_5_D increment is probably related to the inherent characteristics of S_5_D. While triggered AD propagates seizure stages, wide spread propagation of AD induces S_5_D.

Although, attenuation of the GABA inhibitory effect and reduction of the inhibitory post-synaptic potential can cause ADD prolongation, neverthelessthe application of GABA has not changed synaptic responsiveness (32). The rapid EB effect on ADD (0.25 h) and its reversible nature is the evidence for direct non-genomic effects of EB on cell membrane.

To rule out the probability of the involvement of genomic effects of EBon kindling parameters, TAM (an estrogenic receptor antagonist) and anisomycine (a protein synthesis inhibitor) wereapplied ip and intra-amygdala injections respectively. The TAM was applied at low dose (1m/Kg) to inhibit estrogenic receptors without partial effects or antioxidant activity (5). Higher doses of TAM can induce epileptogenic effects as reported previously (20). In addition, TAM pre-treatment could not block ADD and S5D prolongation. Moreover, the inability of anisomycine to inhibit EB induced ADD prolongation confirmed the probable non-genomic rapid effects of EB on ADD.

To facilitate the responsiveness to glutamate, E2 probably either binds directly to a membrane protein, which is partly or totally accompanied by glutamate receptors, or affects glutamate receptors indirectly through disturbing the membrane bilayer lipids (2, 33). This may result from one or a combination of several mechanisms described so far for E2. In addition, the changes in the level or affinity of receptors for NMDA and non-NMDA excitatory amino-acids (3, 30 32) and increased neuronal responsiveness to excitatory amino-acids (25) are probably involved in the late effect of EB. The NMDA receptor antagonist MK-801 may prevent the hormone-induced changes in spine density, NMDA transmission, and long term potentiation (LTP) magnitude (26).

In conclusion, based on rapid EB effects on ADD (0.25 h), its inhibition by Mk801, and inability of anisomycine (in this study) and tamoxifen to prevent AD prolongation, the acute effect of EB on kindled seizures may be induced more via a non-genomic membrane glutamate receptor rather than intracellular estrogenic receptors. The results of this investigation suggest the possibility of the effectiveness of antiepileptic drugs such as topiramate in catamenial epilepsy.
